# Dynamics of a vertical cavity quantum cascade phonon laser structure

**DOI:** 10.1038/ncomms3184

**Published:** 2013-07-25

**Authors:** W. Maryam, A. V. Akimov, R. P. Campion, A. J. Kent

**Affiliations:** 1 School of Physics and Astronomy, University of Nottingham, University Park, Nottingham NG7 2RD, UK

## Abstract

Driven primarily by scientific curiosity, but also by the potential applications of intense sources of coherent sound, researchers have targeted the phonon laser (saser) since the invention of the optical laser over 50 years ago. Here we fabricate a vertical cavity structure designed to operate as a saser oscillator device at a frequency of 325 GHz. It is based on a semiconductor superlattice gain medium, inside a multimode cavity between two acoustic Bragg reflectors. We measure the acoustic output of the device as a function of time after applying electrical pumping. The emission builds in intensity reaching a steady state on a timescale of order 0.1 μs. We show that the results are consistent with a model of the dynamics of a saser cavity exactly analogous to the models used for describing laser dynamics. We also obtain estimates for the gain coefficient, steady-state acoustic power output and efficiency of the device.

## Abstract (web summary)

Phonon lasers are the acoustic equivalent to optical lasers. Here Maryam and colleagues study the dynamics of semiconductor phonon lasers operating in the terahertz frequency regime, and show that these dynamics are similar to that of comparable optical lasers.

## Graphical abstract

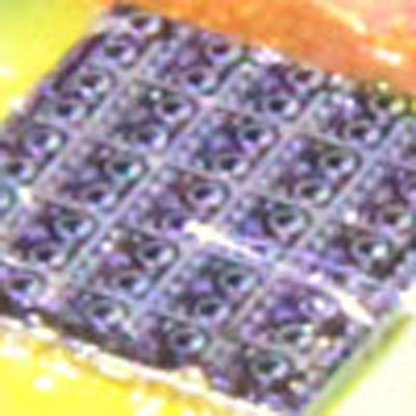

As sources of intense and coherent acoustic waves, sound amplification by the stimulated emission of (acoustic phonon) radiation, saser, devices could find widespread applications in science and technology. For devices operating in the technologically important terahertz (THz) frequency range, applications include sources for acoustic nanoscopy; the generation and manipulation of THz electromagnetic signals; and ultrafast acousto-optic modulation. Potential applications such as these, as well as the fundamental scientific interest in achieving the acoustic equivalent of lasing, have motivated researchers to work towards producing a practical saser device ever since the invention of the maser and laser around 1960.

Recently, experimental demonstrations of sound amplification by the stimulated emission of acoustic phonons at various frequencies have been reported in the literature. Examples include phonon avalanches in optically pumped ruby at 50 GHz (ref. 1); optomechanical devices at megahertz frequencies^2^; optically pumped trapped ions at very low frequencies^3^; and acoustic amplification at about half a THz, due to electron–phonon interactions in semiconductor superlattices (SLs)^4^,^5^. The latter is particularly important as it addresses the generation of coherent acoustic waves in the THz frequency range with corresponding acoustic wavelengths in the nanometre range, useful in a number of applications^6^.

The principle of operation of the SL saser is illustrated in Fig. 1. The saser SL consists of alternating layers of two different materials, in this case GaAs and AlAs, each a few nanometres thick, repeated many times. The electronic band structure of the SL, shown in Fig. 1a, consists of quantum wells for electrons in GaAs, separated by narrow AlAs barriers. The layers are doped so that there are free electrons that can quantum mechanically tunnel through the barriers and occupy an electronic miniband^7^. When a sufficiently large electrical bias is applied to the SL the electronic energy band is strongly ‘tilted’, Fig. 1b, which ‘breaks’ the miniband and the electrons become localized within the quantum wells, forming a Wannier–Stark ladder of states^8^. In this case, electrical conduction through the SL, perpendicular to the layers, is by phonon-assisted hopping, where an electron can jump to a neighbouring well with the emission or absorption of an acoustic phonon to conserve energy^9^. Detailed theoretical calculations of the probabilities of electron transitions involving the emission/absorption of longitudinal polarized acoustic (LA) phonons^10^,^11^, show that, because the transitions are indirect in *k*-space, there is a partial population inversion for phonons of frequency ν≤*Δ*, where *Δ* is the Stark splitting. Under these conditions, the dominance of stimulated phonon-assisted electron tunnelling gives rise to amplification of these LA phonons^12^. Every electron that passes through the SL potentially results in as many stimulated transitions as there are steps in the Wannier–Stark ladder, hence we may call this a ‘quantum cascade’ saser.

Experimental evidence for sub-THz sasing in a distributed feedback (DFB) semiconductor SL device was the observation of a threshold when *Δ* was tuned to a high-Q cavity mode^13^ and acoustic spectral line narrowing^4^. The condition to achieve saser oscillations is that the acoustic losses, which include the wanted sound output, are compensated by the acoustic gain at the frequency of operation. In lasers this is achieved for light by increasing the effective path length in the gain medium by placing it within an optical cavity, commonly a Fabry–Perot cavity formed between a pair of mirrors. Owing to the speed of sound being about five orders of magnitude less than the speed of light, the wavelength of THz phonons is of order nanometres compared to millimetres for THz photons. Furthermore, the scattering of THz phonons is generally much stronger than for photons. Therefore, the requirements for an acoustic cavity are more critical than for a THz optical cavity. However, efficient acoustic microcavities formed between a pair of SL acoustic Bragg mirrors have been demonstrated in a number of experiments^14^,^15^,^16^, which suggests that SL Bragg mirrors could be used to make an efficient saser acoustic cavity.

In the work described here we combine in a single structure a SL gain medium and SL acoustic Bragg mirrors to form the basis of an electrically pumped vertical cavity saser device. Using superconducting bolometers opposite the device, we measure the acoustic phonon emission of the device as a function of the electrical pump amplitude, *V*, and the time, *τ*, after turning on the pump. At sufficiently high *V*, the emission builds up in intensity, initially exponentially with time, and reaches a steady state on a timescale of the order 0.1 μs. We show that the results are consistent with a theoretical model of the dynamics of a saser cavity, which is analogous to the models used for describing the build-up of light intensity after turning on a laser. Fitting the model to the data provides estimates for the gain coefficient of the amplifying SL, the steady-state acoustic power output and the efficiency of the device.

## Results

### Experimental device structure

**Figure 2:**
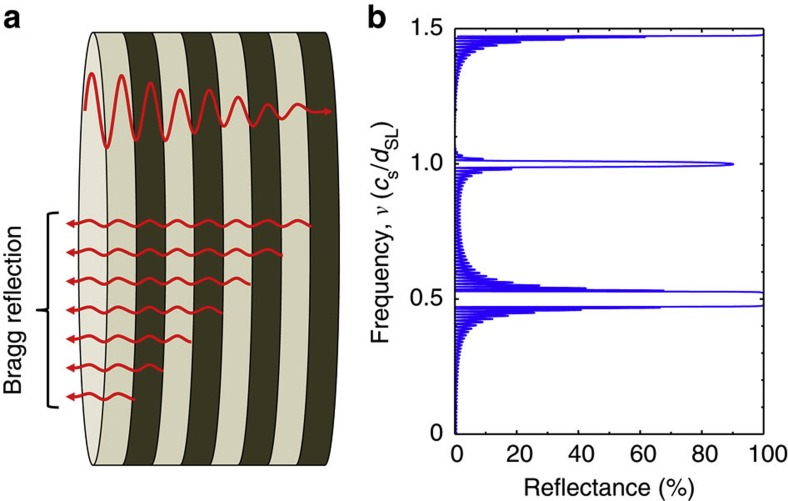
Superlattice acoustic Bragg mirror: (**a**) incident acoustic wave is partly reflected at each interface due to the difference in the acoustic impedances of the materials making up the layers of the superlattice. If these partial reflections are in phase, strong reflection occurs; (**b**) calculated reflectance of a 40-period GaAs/AlAs SL with equal thicknesses of GaAs and AlAs as a function of the frequency in units of *c*_s_/*d*_SL_.

The working of a SL acoustic Bragg mirror is illustrated in Fig. 2. The two different materials making up the SL have slightly different acoustic impedances, which results in an acoustic wave incident on an interface between them being partially reflected and partially transmitted, Fig. 2a. If the total thickness of a pair of layers, known as the SL period, *d*_SL_, is appropriately related to the wavelength, λ, of the sound, for example, for normal incidence: *n*λ=2*d*_SL_, where *n* is an integer, the partial reflections from many such layers add in phase giving rise to the Bragg reflection. A transfer matrix technique, analogous to that used for multilayer dielectric optical mirrors, may be used to calculate the acoustic reflectance of a SL^17^. Figure 2b shows the calculated normal incidence reflectance for LA phonons as a function of frequency, *ν*, for a Bragg mirror SL made from 40 pair of GaAs and AlAs layers of equal thickness, *d*_SL_/2. It is seen that at the lowest frequency of Bragg reflection, *ν*=*c*_s_/2*d*_SL_ (*c*_s_ is the speed of sound), the reflectance is nearly 100%.

**Figure 3:**
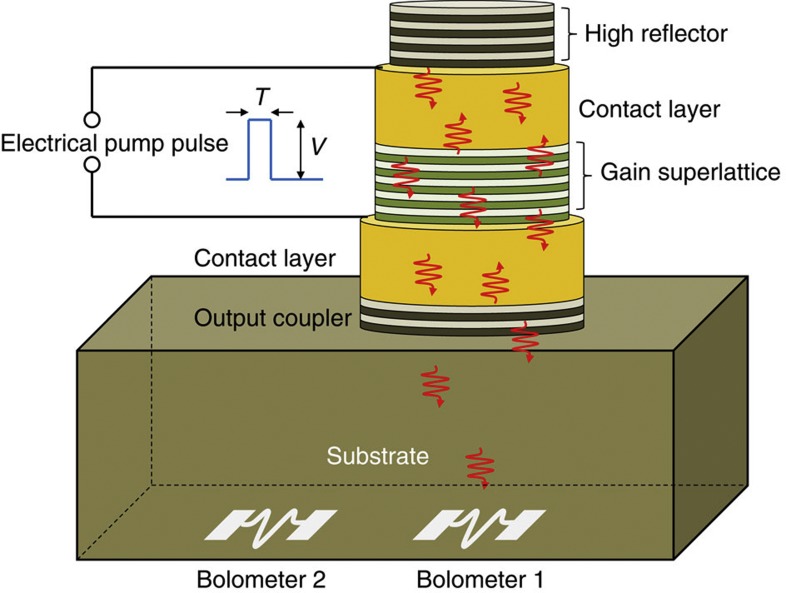
Schematic of vertical cavity saser device: the device is based on a structure consisting of a semiconductor superlattice gain medium in a cavity between two superlattice acoustic Bragg mirrors. The top, high reflector, mirror was designed to have a reflectance *R*_1_=100% and the bottom, output coupler, mirror a reflectance *R*_2_=97% both at 325 GHz. On the opposite side of the substrate are two superconducting bolometers for detecting the acoustic output.

Figure 3 shows the experimental device structure consisting of the phonon amplifying (gain) SL and, at each end of it, the SL Bragg reflectors. The design frequency of the structure is 325 GHz, determined by the period of the mirror SLs. Superconducting bolometers fabricated on the opposite side of the substrate detect the phonon emission. One bolometer is positioned directly opposite the centre of the device and the other subtends an angle of about 57 degrees to the normal to the device structure.

### Time-integrated signals

**Figure 4:**
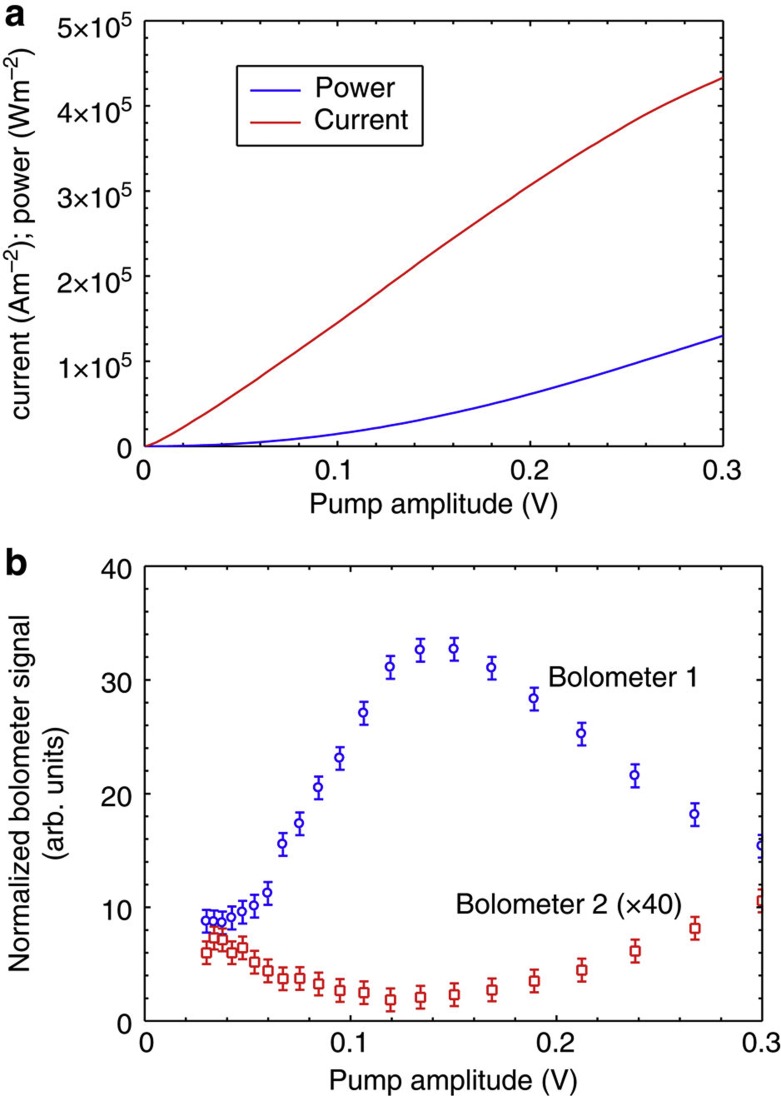
Device characteristics and time-integrated signals: (**a**) current–voltage (*I–V*) and power–voltage (*P–V*) characteristics of the gain SL; (**b**) time-integrated signals on bolometer 1 (directly opposite the vertical cavity structure) and bolometer 2 (making an angle of 57 degrees to the central axis of the structure) as a function of the electrical pump pulse amplitude. The signals have been normalized to the total power dissipated in the device at each value of the applied pump pulse amplitude. Error bars indicate the s.d. of the bolometer signal measurements.

Figure 4b shows the time-integrated bolometer signals as a function of the pump voltage *V* for pump pulse duration *τ* of 150 ns. The signals have been normalized to the total power dissipated in the device deduced from current–voltage measurements, as shown in Fig. 4a. At bolometer 1, directly opposite the device, there is a clear broad peak centred near *V*=140 mV. This indicates that, in a range of pump amplitudes around 140 mV, a larger fraction of the total power emitted as phonons is reaching bolometer 1.

No peak is observed at bolometer 2. In fact, there is a clear dip over the range of pump amplitude where the peak is seen at bolometer 1. Bolometer 2 is located at a position where it detects mainly phonons that are emitted at large angles (≥57 degrees) to the axis of the acoustic cavity. Thus the increase in the phonon emission in the forward direction, detected by bolometer 1, appears to be accompanied by a decrease in the emission at larger angles.

### Temporal dependence of the signals

**Figure 5:**
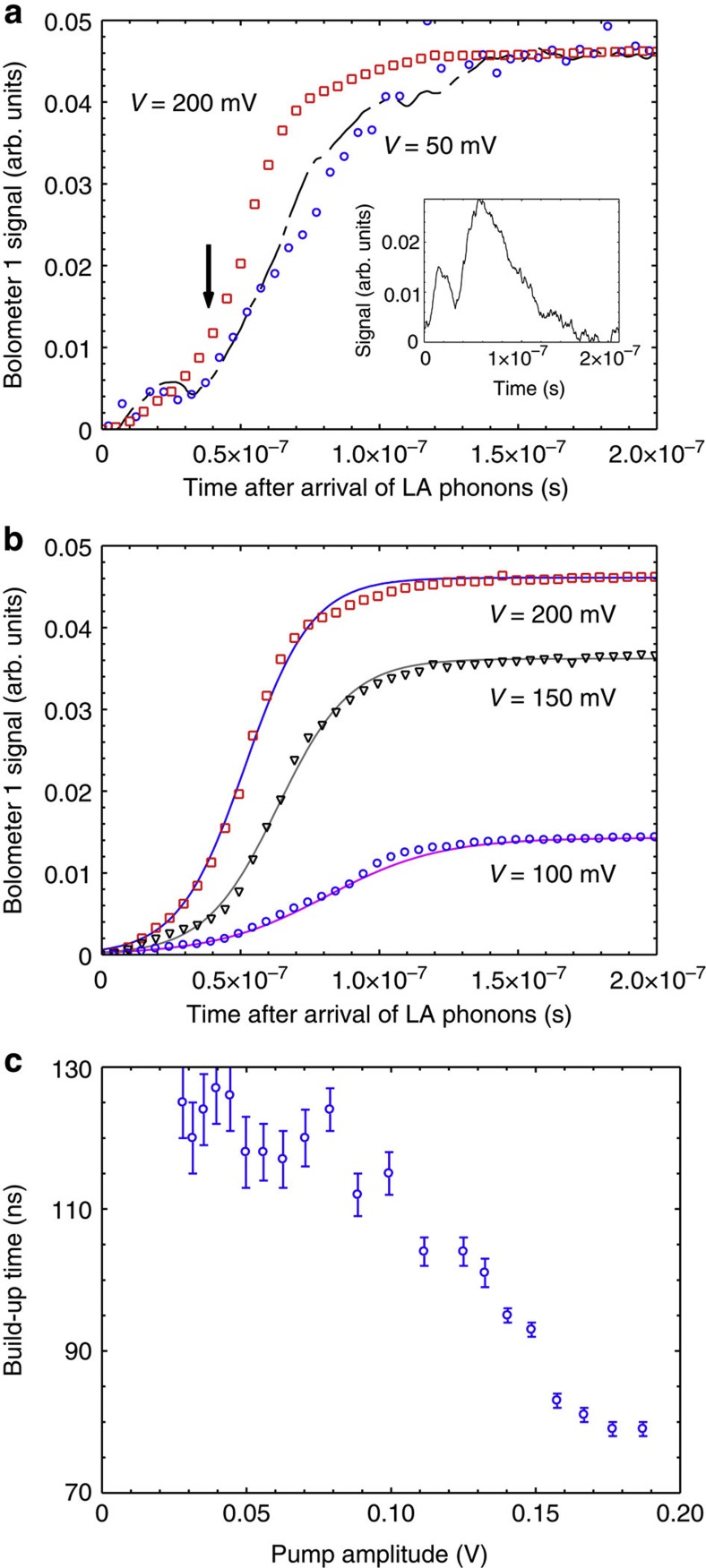
Temporal dependence of the signals: (**a**) signals at bolometer 1 for two different amplitudes of the 200 ns-duration pump pulse, one above and one below *V*=80 mV. Also shown (dashed line) is the simulated build-up based on convolving the signal from a 10 ns-duration pump pulse (shown in the inset) with a 200 ns rectangular pump pulse. The arrow indicates the expected time of arrival at the bolometer of transverse-polarized phonons. The traces are normalized to the steady-state value of the 200 mV signal; (**b**) build-up of the LA signal for three pump amplitudes above *V*=80 mV, the solid lines are fits of Equation 2 to the data assuming saser action is taking place; (**c**) time for the LA signal to build-up to 90% of its steady-state value, which is reached at long times (>150 ns). Error bars indicate the s.d. of the bolometer signal measurements.

To study the dynamics of the phonon emission we first compare the time evolutions of the bolometer 1 signals *I*_*V*_(*t*) measured at two pump amplitudes, one at *V*=50 mV, below the value where the increase in the normalized bolometer 1 signal occurs (≈80 mV), and one at *V*=200 mV, which is past the peak in the normalized bolometer 1 signal. The symbols in Fig. 5a show the normalized temporal traces measured for pump pulse durations *τ*=200 ns. Zero on the time axis corresponds to the time at which the first LA phonons (speed≈4,800 ms^−1^) reach the bolometer. Both signals show a temporal build-up after which a steady-state level is reached. The two temporal traces are normalized to the steady-state level. It is clearly seen that, at *V*=200 mV, build-up is more rapid and the steady-state level is reached earlier than for the lower pump amplitude. This experimentally observed difference is the main result of the present work and, in the discussion section below, we show that this is consistent with the assumption that the device is working as a saser.

Figure 5b shows the build-up of the signal on bolometer 1 for 200 ns-duration pulses of different amplitude (all above *V*=80 mV). We see that the signal builds up with time reaching a steady state in about 80 ns for *V≥*150 mV. At lower values of pump amplitude, the time to reach steady state is longer, about 100 ns for *V*=100 mV. Defining the build-up time as the time for the signal to reach 90% of the steady-state value, Fig. 5c shows the build-up time as a function of the pump amplitude. It is interesting to compare Fig. 5c with Fig. 4b: at the pump amplitudes where the peak in Fig. 4b occurs, the build-up time for the signal at bolometer 1 is shorter than at smaller pump amplitudes.

## Discussion

Considering just the wanted output through the output coupler Bragg mirror SL (reflectance, *R*_2_=97%), the acoustic loss per round trip of the acoustic cavity is 3%. The maximum gain for LA phonons when the Stark splitting is approximately equal to the phonon energy may be calculated^5^,^10^,^11^, and for 325 GHz phonons is about 10% per round trip. Therefore, even after allowing for the uncertainty in the gain and additional losses due to phonon scattering in the cavity, the vertical cavity structure we have made is theoretically capable of achieving and sustaining phonon oscillation when pumped above a threshold. Allowing for the fact that a fraction of the pump voltage is dropped across the device contacts, 140 mV pump amplitude corresponds to a Stark splitting, *Δ*, which is appropriate for amplification of 325 GHz phonons. We therefore attribute the peak in the normalized signal at bolometer 1, Fig. 4b to the tuning, by changing the pump amplitude, of the maximum of the gain spectrum into resonance with the energy of the cavity-confined phonon modes, thus satisfying the saser threshold condition^13^. The width of the peak suggests that this threshold condition is satisfied for pump amplitudes in the range about 80–250 mV. This breadth of gain spectrum is probably due to disorder in the gain SL^5^.

The dip in the normalized signal at bolometer 2, Fig. 4b, suggests that the increase in the phonon emission in the direction of bolometer 1 could be at the expense of phonon emission at larger angles. In the case of lasers, light emission in directions other than along the axis of the cavity is referred to as ‘sidelight fluorescence’ and the component due to non-lasing emission is observed to decrease when the threshold for lasing is achieved^18^. Our observations using bolometer 2 are consistent with the acoustic equivalent of this behaviour.

In the analysis of the temporal traces of *I*_*V*_(*t*), we consider the signal build-up under the conditions when the saser effect is expected to be present and in the trivial case when no phonon-stimulated emission takes place. For the latter, the signal at bolometer 1 may be written as a temporal convolution:


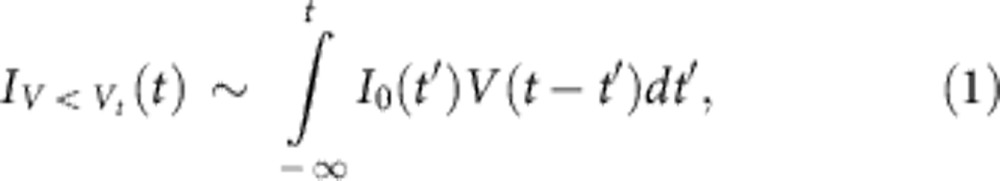


where *I*_0_(*t*) is a bolometer signal for infinitively narrow excitation pulse at the SL, and *V*(*t*) is the temporal evolution of the excitation pulse. The signal *I*_0_(*t*) will be determined by such factors as: the rate of spontaneous emission of phonons by electrons in the SL; geometrical factors due to the size of the phonon source and detector giving a spread of flight times; and the finite response time of the bolometer and detection electronics. It consists of two short pulses corresponding to LA and also transverse-polarized acoustic (TA) phonons. The dashed line in Fig. 5a was obtained by convolving the phonon signal from a 10 ns-duration pulse (shown in the inset to Fig. 5a) with the 200 ns-long rectangular pump pulse, and normalizing to the same steady-state values as the other two curves. It is clear that this accounts fairly well for the growth of the signal for *V*=50 mV pump amplitude including a distinct feature (indicated by the arrow) at around 40 ns after the start of the signal when the TA phonons (speed≈3,200 ms^−1^) are expected to arrive at the bolometer. For the pump amplitude *V*=200 mV, the feature at 40 ns is suppressed meaning that TA phonons make a much less significant contribution than LA. This is consistent with theoretical predictions that phonon amplification in the electrically pumped SL is effective for LA phonons, which are more strongly coupled to the electrons via the deformation potential than the TA modes^10^,^11^. Therefore, in the following analysis of the signals for *V*>80 mV, the TA modes will not be considered further.

We attribute the increase with time of the bolometer signal for *V*>80 mV pumping to the build-up of phonon oscillations in the device’s acoustic cavity. Now consider the build-up with time of the signal at pump amplitudes, which satisfy the saser threshold condition: qualitatively, the build-up time is given by the time taken for phonons to make one round trip of the cavity multiplied by the number of round trips required for the intensity to reach a level where the gain is reduced due to depletion of the available population inversion. The latter depends on the net small-signal gain (gain–losses) per round trip. Following the method used to calculate the build-up time of the optical emission of lasers^19^, we arrive at the following quantitative expression for the time dependence of the LA phonon intensity in the cavity


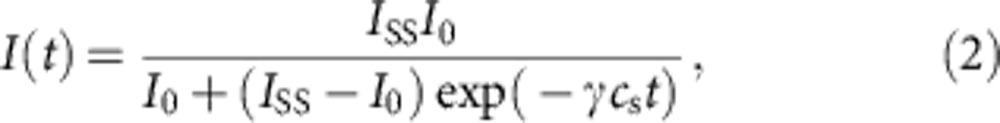


where *I*_0_ is the initial intensity at the instant the build-up starts; *I*_ss_ is the steady-state intensity reached at ‘long’ times, after the build-up is complete; *γ* is the net gain per unit length and *c*_s_=4,800 ms^−1^ is the speed of LA phonons. Equation (2) is of course only valid if the threshold for saser oscillation is achieved, that is, *γ* is positive. The two contributions to the losses are the wanted acoustic output through the output coupler mirror and phonon scattering inside the cavity. For the latter we have considered scattering by: structural defects; mass defects, including isotopes and impurities; and scattering by free electrons in the highly doped contacts to the amplifying SL. For the first two, we estimate that the mean free path of 325 GHz phonons in good quality GaAs at low, liquid helium, temperatures is of order millimetres^20^, and as the cavity length is only 1.5 μm these may be neglected. Scattering of phonons by free conduction electrons has been considered by Ziman^21^. Following this approach, we estimate the mean free path in the contact layers is about 60 μm for 325 GHz phonons, which gives a loss per round trip of 3% which certainly cannot be ignored. Therefore, we have for the net gain per unit length


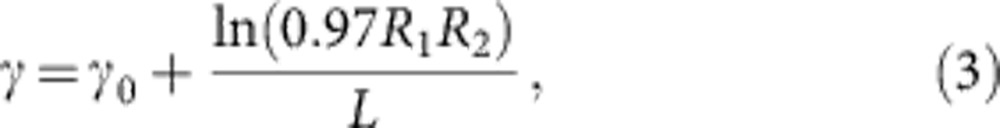


where *γ*_0_ is the small-signal gain of the amplifying SL at low intensity (below saturation); *R*_1_ and *R*_2_ are the SL Bragg mirror reflectances; and *L* is the cavity length. The solid lines in Fig. 5b are the result of fitting equation (2) to the experimental data, a very good fit is obtained using the parameters *I*_ss_/*I*_0_ and *γ* listed in Table 1. The quality of the fit supports our interpretation that the increase of the bolometer signals with time maybe due the build-up of oscillations in the device when the pump amplitude satisfies the threshold condition (*V*≥80 mV).

From the values of *γ*, *R*_1_*R*_2_=0.97 and using equation (2), we can obtain the gain coefficient, *γ*_0_, of the amplifying SL at 325 GHz for each different pump amplitude, and the values are listed in Table 1. The maximum value of *γ*_0_, at pump amplitude 200 mV, is about an order of magnitude smaller than the measured gain of a similar SL at 650 GHz^5^. This difference can be largely attributed to the phonon frequency dependence of the transition rates in a realistic, disordered, system^5^ and at finite temperature^10^, which are larger for higher frequency phonons.

To estimate the acoustic output under saturation conditions we use the values of the parameter *I*_ss_/*I*_0_ obtained from fitting equation (2) to the data. Assuming that the build-up of phonon oscillations in the cavity starts when the stimulated emission rate becomes equal to the spontaneous emission rate, *I*_0_ corresponds to the situation where there is one phonon per mode in the cavity. The density of phonon states per unit volume per unit frequency interval is given by


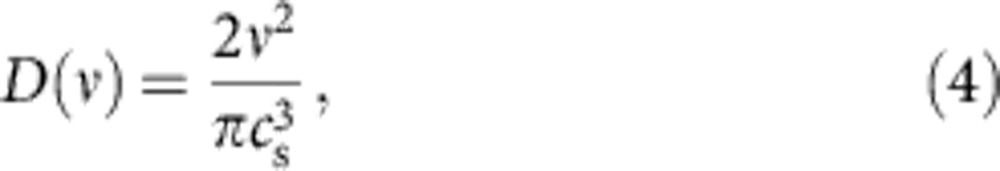


where *v* is the frequency. Therefore


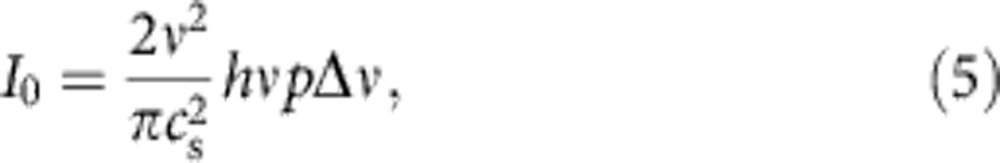


where *p* is the number of longitudinal cavity modes falling inside the reflectance band of the mirrors, which is 30 GHz-wide, and Δ*v* is the linewidth of a single longitudinal cavity mode, given by Δ*v*=1/2*πτ*_cav_, where *τ*_cav_ is the cavity lifetime. The length *L* of the cavity is ≈1.5 μm, therefore the frequency spacing between the longitudinal cavity modes *c*_s_/2*L*≈1.7 GHz, which gives *p*≈18. The cavity lifetime is related to the time to make one round trip in the cavity, *τ*=2*L*/*c*_s_, and the loss per round trip


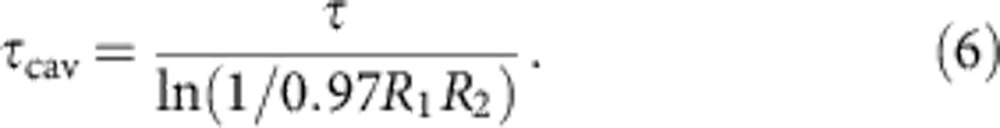


Thus, evaluating equation (5) gives *I*_0_=176 Wm^−2^. Using the values of *I*_ss_/*I*_0_ in Table 1, we can determine the steady-state intensity inside the cavity *I*_ss_, and allowing for the transmission of the output coupler, *T*_2_=1−*R*_2_=0.03, corresponding values of acoustic output, *P*_o_, are deduced and presented in Table 1. Comparing the values of *P*_o_ with the electrical power consumed by the device at the different values of *V*, we may estimate the efficiency, *η*, of the device listed in Table 1, which is of the order a few percent. The efficiency drops for pump amplitudes above about 150 mV, even though the value of the gain is larger, because the contribution of the power dissipated in the device contact resistances increases as the square of the device current.

In conclusion, we have fabricated a vertical cavity structure based on semiconductor (AlAs/GaAs) SLs as the acoustic gain medium and the acoustic Bragg mirrors, which is designed to operate as a saser oscillator device at 325 GHz. Following turning on of the electrical pumping to the device, we observe a build-up of the acoustic emission in a direction parallel to the axis of the acoustic cavity on a timescale of the order 0.1 μs. This is accompanied by a reduction in the ‘side’ emission from the cavity. These measurements are consistent with the device operating as a saser oscillator when electrically pumped above a threshold of 80 mV. Measurement of the phonon emission dynamics allowed us to determine the gain coefficient and the acoustic power output at a frequency of 325 GHz. We obtained *γ*_0_ in the range 5.0–5.8 × 10^4^ m^−1^ and *P*_o_ in the range 285–670 Wm^−2^, depending on the amplitude of the applied pump pulse. The advantage of this vertical cavity device compared to the DFB structure previously investigated is that the frequency of operation can be engineered by changing the parameters of the acoustic Bragg mirrors, and thus can be matched with the requirements of any application. In the DFB saser, the frequency of operation was fixed by the parameters of the gain SL necessary to achieve the optimum amplification. However, our measurements show that the effect on the phonon oscillation build-up time of the increased length of the cavity in the vertical cavity structure needs to be taken into account when considering applications requiring short (≤10 ns) acoustical pulses.

## Methods

### Device structure and fabrication

The device structure consisting of a semiconductor SL gain region and acoustic Bragg mirrors was grown by molecular beam epitaxy on a 380-μm-thick semi-insulating GaAs substrate. A single period of the 50 period gain SL consists of 6 nm of GaAs and 4 nm of AlAs, both n-doped with Si to a density of 10^17^ cm^−3^. At each end of the SL was a 0.5 μm, n^+^-doped (10^18^ cm^−3^ of Si) electrical contact layer. SL acoustic Bragg mirrors were fabricated at both ends of the gain SL: a single period of the mirrors consists of 4 nm of GaAs and 4 nm of AlAs, both undoped. The top mirror (high reflector) consisted of 40 periods and was designed using the transfer matrix method to give near 100% reflectance in a 30-GHz bandwidth, centred on 325 GHz. The bottom (substrate end) ‘output coupler’ mirror was 15-periods giving a reflectance ~97% at 325 GHz. Using conventional photolithographic semiconductor processing techniques, the vertical cavity saser structure was processed into a 400-μm-diameter device and ohmic contacts formed to the ends of the gain SL.

### Bolometer fabrication and measurements

The acoustic emission was measured using two superconducting granular aluminium bolometers deposited by vacuum evaporation on the reverse side of the GaAs substrate to the device. One bolometer was placed directly opposite the centre of the device, and the second was placed just off the edge of the device, see Fig. 3. The active part of the bolometer was defined photo lithographically: it was a 30-nm-thick strip of aluminium 100 μm in length and 10 μm in width, shaped into a loop fitting within a 40-μm square area. The aluminium was evaporated from an alumina crucible to incorporate impurities in the film and raise the transition temperature to about 2 K. At room temperature the resistance of the bolometer was measured to be ≈200 Ω. The experiments were carried out at a temperature of 2 K, which is on the superconducting transition edge of the bolometer and where its resistance was about 50 Ω. Under these conditions, the electrical resistance of the bolometer was highly sensitive to the acoustic flux falling on it. The device was excited using electrical pump pulses of variable duration, *τ*, from 10 ns to 0.2 μs and amplitude, *V*, from 0 to 300 mV. The bolometer was biased at a constant current of 10 μA, and the voltage signal was amplified 100 × using 1-GHz bandwidth electronics and recorded using a fast real-time digital sampling oscilloscope.

## Author contributions

A.J.K. designed the experiments and analysed the data. W.M. and A.V.A. performed the experiments and analysed the data. R.P.C. fabricated the device structures. A.J.K., W.M. and A.V.A. prepared the manuscript.

## Additional information

**How to cite this article:** Maryam, W. *et al*. Dynamics of a vertical cavity quantum cascade phonon laser structure. *Nat. Commun.* 4:2184 doi: 10.1038/3184 (2013).

## Figures and Tables

**Figure 1:**
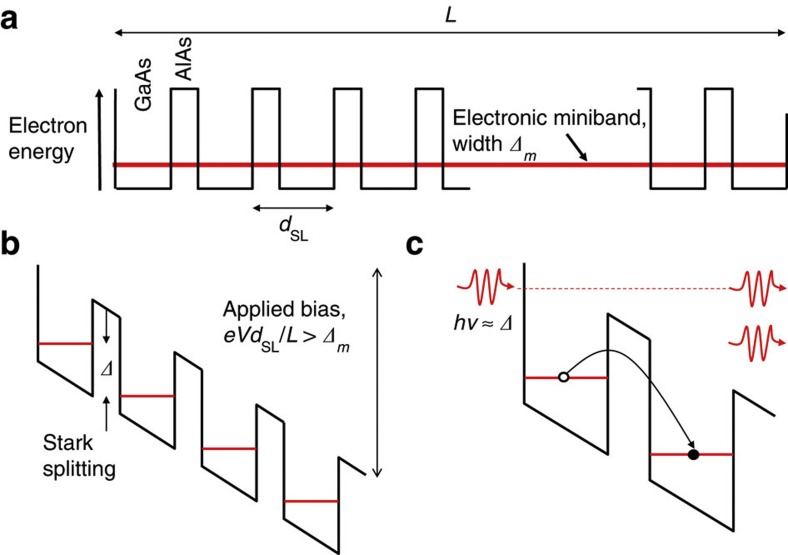
Electronic energy band structure of the gain superlattice: (**a**) under zero-applied electrical field; (**b**) under an applied electric field sufficiently large to form a Wannier–Stark ladder; (**c**) illustration of phonon-assisted (with stimulated emission) hopping of electrons between neighbouring quantum wells.

**Table 1:** Device characteristics.

**Pump amplitude**, ***V*** **mV**^**−1**^	***I***_**ss**_**/*****I***_**0**_	***γ*** **m**^**−1**^	***γ***_**0**_ **m**^**−1**^	***P***_**o**_ **Wm**^**−2**^	***η*** **%**
100	54	9.8 × 10^3^	5.0 × 10^4^	285	10
150	127	1.5 × 10^4^	5.6 × 10^4^	670	11
200	74	1.7 × 10^4^	5.8 × 10^4^	390	4
